# Adsorption of Organosilanes on the Surface of Aluminium and the Formation of Organosilane Films to Protect It from Corrosion

**DOI:** 10.3390/ma14195757

**Published:** 2021-10-02

**Authors:** Natalia Gladkikh, Maxim Petrunin, Ludmila Maksaeva, Tatyana Yurasova

**Affiliations:** Frumkin Institute of Physical Chemistry and Electrochemistry, Russian Academy of Sciences, 119071 Moscow, Russia; mmvp@bk.ru (M.P.); lmaksaeva@mail.ru (L.M.); tatal111@yandex.ru (T.Y.)

**Keywords:** adsorption, aluminium, organosilane films, corrosion

## Abstract

Adsorption of diaminesilane (DAS), vinyltrimethoxysilane (VS) on the surface of thermally precipitated aluminium was examined. The use of different adsorption isotherms made it possible to calculate the adsorption heats for DAS and VS. It was determined that chemisorption of these organosilanes occurred on the surface of aluminium. Exposure of aluminium for 60 min to aqueous solutions of organosilanes led to the formation of organosilane films on the surface of the metal. The use of infrared spectroscopy and scanning electron microscopy in the work made it possible to assess the interactions of organosilanes with the metal surface, as well as to determine the structural features of the films and their thickness. Electrochemical and corrosion research methods made it possible to study the protective properties of organosilane films on aluminium.

## 1. Introduction

Aluminium (Al) and its alloys are used in many applications because they combine lightness with strength. They have high thermal and electrical conductivity, reflect light, and are hygienic and nontoxic. Such applications include aircraft engineering, mechanical engineering, shipbuilding, and energetic uses. Materials based on Al are produced in a wide variety of forms according to their different uses [1]. At the same time, while being stable under the conditions of uniform corrosion, they have low resistance to corrosion in chloride-containing environment and are prone to pitting corrosion [2]. Pitting corrosion is one of the most dangerous types of metal structure damage; it occurs on metals covered with an oxide film at locations with passive film damage [2,3]. Owing to high metal dissolution rates in local areas, pitting corrosion may eventually result in the failure of the entire structure. Despite efforts to overcome pitting corrosion of aluminium and its alloys over several decades [4,5,6], the development of effective methods to mitigate the pitting corrosion of Al and its alloys is still relevant.

In general, materials based on aluminium and its alloys are protected from corrosion during their usage by means of polymer and paint coatings. Such coatings have been well-established as one of the most effective ways to decrease corrosion risk under various conditions [7,8,9,10,11,12,13,14]. Often, for the application of coatings, the metal surface is subjected to a preliminary chemical treatment (finishing). This improves its corrosion resistance and increase the adhesion of the paint (polymer) coating onto the metal. Until recently, the metal surface was chemically treated with hexavalent chromium compounds. Chromium layers effectively protect the metal from corrosion, while facilitating high adhesion to protective coatings. However, hexavalent chromium is an environmentally harmful compound and its use is currently highly restricted, and it is likely to be completely banned in the future [15]. In recent years, research efforts have been aimed at replacing the chromium-based technologies with other, more benign processes, particularly in the processing of Al and its alloys [16]. However, the issue remains unresolved, and the development of more efficient and environmentally friendly methods for pretreating aluminium surfaces is an urgent scientific and technical task.

Organosilicon compounds, in particular organosilanes, are most often used as an alternative to hexavalent chromium substances. Organosilanes are compounds containing a Si atom in the molecule, which is connected to the C atom directly or through O atoms: R_n_SiX_4−n_ (n = 1–4, R is an organic radical, X is H, Cl, Br, OH, OR and other functional groups) [17,18]. Organosilanes are artificial compounds that are synthesized from silicon dioxide [19]. Recently, this class of organosilicon compounds has been used as a metal corrosion inhibitor [3,7,8,9], including for pretreatment of Al surface from local dissolution [15]. This is due to its ability to adsorb on metal surfaces with the formation of siloxane structures resulting from polycondensation [20,21,22]. It has been reported in some papers [8,23,24,25,26] that the presence of organosilanes such as 3—aminopropyltriethoxysilane, 3—glycidoxypropyltrimethoxysilane, ureidopropyltriethoxysilane, vinyltrimethoxysilane on metals promotes resistance to aggressive corrosive environments. However, even with modification of the surface of Al with organosilanes for its protection, research on the adsorption of silanes on aluminium in the presence of corrosive media remains relevant. As such, the purpose of this work was to examine the adsorption of diaminosilane and vinyltrimethoxysilane on Al as well as study the structural and protective properties of organosilane films on its surface.

## 2. Materials

### 2.1. Material for Adsorption Studies

To study the adsorption processes on the surface of aluminium, thermally precipitated aluminium was used on a quartz substrate [27]. For this, the aluminium was hung in a vacuum evaporator, VUP-5 (“V-KIP”, Belgorod, Russia), to heat the metal to the evaporation temperature by means of transmitting a current of 18 A at a pressure of 10^−6^ mm. Hg. The substrate was a quartz resonator of the brand QC-10-AuBU (“Wurth Elektronik”, Waldenburg, Germany) with gold plating, an AT section, and a base frequency of 10 MHz. The surfaces of the substrates were degreased with alcohol, washed with water, and dried in the air at room temperature.

To study adsorption on thermally precipitated aluminium, two organosilanes (“Witco Co.”, Geneva, Switzerland) were used:

1. Vinyltrimethoxysilane (VS) C_5_H_12_O_3_Si
2. Aminoethylaminopropyltrimethoxysilane—diaminesilane (DAS) C_8_H_22_N_2_O_3_Si

The concentration of organosilanes was changed in the range of 1 × 10^−5^ to 1 × 10^−3^ M.

### 2.2. Material for Electrochemical and Corrosion Studies

To study the corrosion–electrochemical behaviour of the metal in various aggressive conditions. Aluminium foil of the A995 brand with a thickness of 200 microns was used. Before the studies, the samples were washed using ultrasound in an ultrasound sapphire-0.8 TC (“Sapphire”, Moscow, Russia), in a mixture of C_2_H_5_OH:C_7_H_8_ = 1:1 for 15 min [3,8].

#### Application of Organosilane Films to Aluminium Foil

To investigate the corrosion–electrochemical behaviour of the formed films on Al foil, the organosilanes described in Section 2.1 were used with a concentration of 0.1 M. The base of the organosilane compositions was distilled water, to which organosilanes of various concentrations were added. The resulting solution was subjected to ultrasonic treatment for 15 min. Then, the aluminium foil that was prepared according to Section 2.2 was incubated in the inhibitory solution for 60 min. After that, the samples were dried at T = 120 °C for 30 min. Next, the films on the aluminium surface were examined by physicochemical methods.

### 2.3. Piesoquartz Nanobalance

The Al layer thickness on the samples (prepared according to Section 2.1) was determined using piezoquartz nanoweights [28] with the EQCN700 unit (“Elchema”, Potsdam, NY, USA), by recording and changing the frequency of the quartz resonator in the process of sputtering. After the frequency reached steady-state (i.e., reached a constant value), the calculated amount of organosilane was added to the cell. Then, the frequency change of the resonator was measured. The change in frequency was then recalculated into the change in mass using the Formula (1):

(1) $$\Delta m=-\left(N\rho S\times \frac{\Delta f}{\Delta f_{0}^{2}}\;\right)$$

where Δ*m* is the change of organosilane mass (g); *f*_0_ is the basic base frequency of 10,000 kHz; Δ*f* is the change in frequency of piezoquartz resonator (kHz); *N* is the frequency constant (1670 kHz mm); *ρ* is the quartz density, equal to 2.65 g/cm^3^; and *S* is the quartz working area (0.72 cm^2^).

The resulting layer had a thickness of 1.2 ± 0.1 μm. After the end of deposition, the working volume was pumped out in a vacuum for 30 min, after which the air was started, and the samples were taken out and placed in an exicator with dried CaCl_2_. Adsorption was studied 15 h after metal deposition.

### 2.4. Infrared Spectroscopy

Infrared spectra of organosilane films on the surface of the aluminium were recorded on an IR microscope Hyperion 2000 (“Bruker”, Bremen, Germany), coupled with a vacuum IR Fourier spectrometer IFS-66 v/s (“Bruker”), with a resolution of 2 cm^−1^ in the range of 600–4000 cm^−1^. Processing of spectra was carried out using the software OPUS (“Bruker”), with correction using the Kramers–Kronig transform performed automatically. 

### 2.5. Scanning Electron Microscopy (SEM)

The thickness of the formed films (the interface of Al/organosilane films was investigated by examining the interface cross-sections) and their structure (the presence of porosity) were determined using scanning electron microscopy (SEM). For this purpose, a scanning electron microscope, VEGA 3 SB (“Teskan”, Brno, Czech Republic), equipped with an energy dispersion (EDS) prefix (“Kevex–Ray”, Burlingame, CA, USA) was used.

### 2.6. Corrosion–Electrochemical Studies

#### 2.6.1. Electrochemical Tests

The electrochemical behaviour of the aluminium foil was investigated under conditions of anodic polarization. To remove anode potentiodynamic curves, the potentiostat IPC PRO–MF (“Volta”, Saint Petersburg, Russia) was used, as well as a three-electrode cell with separated electrode spaces. The auxiliary electrode was a platinum plate with an area of 1 cm^2^. A silver chloride electrode was used as a comparison electrode. The working electrode was composed of aluminium plates, with a working area of 2 cm^2^. The background electrolyte was a borate buffer solution of 0.4 M H_3_BO_3_ + 0.1 M Na_2_B_4_O_7_ with a pH of 6.7, containing 0.01 M NaCl. After placing the samples in the cell, they were incubated in the background solution for 30 min until the E_cor_ was established. Then, potentiodynamic curves were taken from the steady-state corrosion potential, with a potential sweep rate of 0.4 mV/s. All potential values are given with respect to the silver chloride reference electrode.

The effectiveness of the formed films was evaluated by changing the potential of local depassivation ∆E in accordance with Formula (2):

(2) $$\Delta E_{pit}=E_{pit}^{inhibitor}-E_{pit}^{background}$$

where $E_{pit}^{inhibitor}$ и $E_{pit}^{background}$ were determined from anodic potentiodynamic curves in a solution with an inhibitor and without one, respectively.

#### 2.6.2. Corrosion Tests

Corrosion tests were carried out in the climatic chamber Terchy (“Terchy Environmental Technology LTD”, Taipei, Taiwan) at a temperature of 60 °C and humidity of 98%. The area of the samples was 9 cm^2^. The time before the appearance of the first defect τ [29] and the proportion of corrosion damage Q [30] were determined. The exposure time of the samples was 720 h.

## 3. Results

### 3.1. Adsorption of Organosilanes

Adsorption of organosilanes on the surface of the aluminium was estimated by changing the frequency of the quartz resonator after introduction into the solution, and calculating the change in mass [28,31].

**Figure 1:**
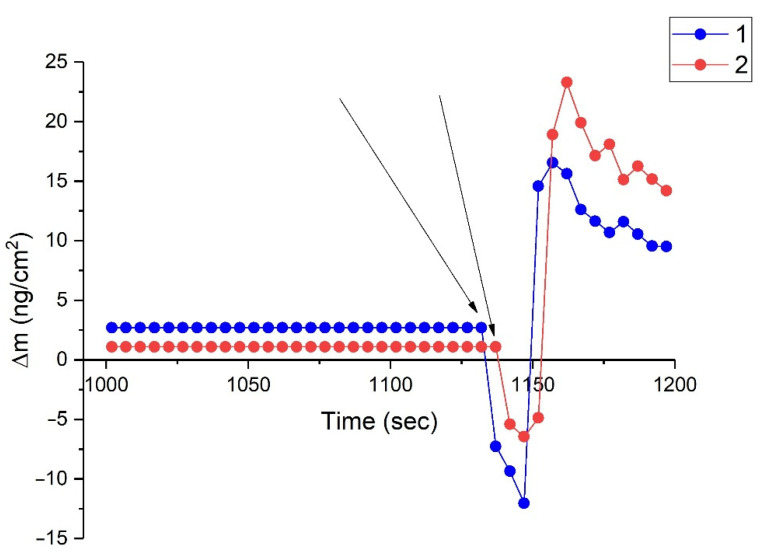
Kinetics of organosilanes adsorption on the surface of freshly sprayed aluminium from aqueous solution: (**1**) DAS; (**2**) VS. **↓** addition of organosilanes to aqueous solution to concentration equal 1 × 10^−5^ M.

The addition of a small amount of DAS in water, corresponding to a concentration of 1 × 10^−5^ M, first led to a slight decrease in the mass of the sample, followed by an increase in mass (Figure 1, curve 1). Similarly, the introduction into the solution of VS at the same concentration led to an initial decrease in the mass of the sample (Figure 1, curve 2). The decrease in the mass of the sample was insignificant at 17.5 and 24.7 ng/cm^2^ for DAS and VS, respectively. Similar effects have previously been observed on copper [4] and have also been observed in the adsorption of corrosion inhibitors on the surface of iron and gold [31]. 

Increasing the concentration of organosilanes in solution led to an increase in adsorption. It was found that the sorbed phase of DAS and VS consisted of reversible and irreversible parts. The reversible adsorbed layer was removed from Al when the sample was soaked in water for 10 min and amounted to about 10% of the total amount of adsorbate. The irreversibly sorbed part of the organosilanes was resistant to water for at least 8 h. 

**Figure 2:**
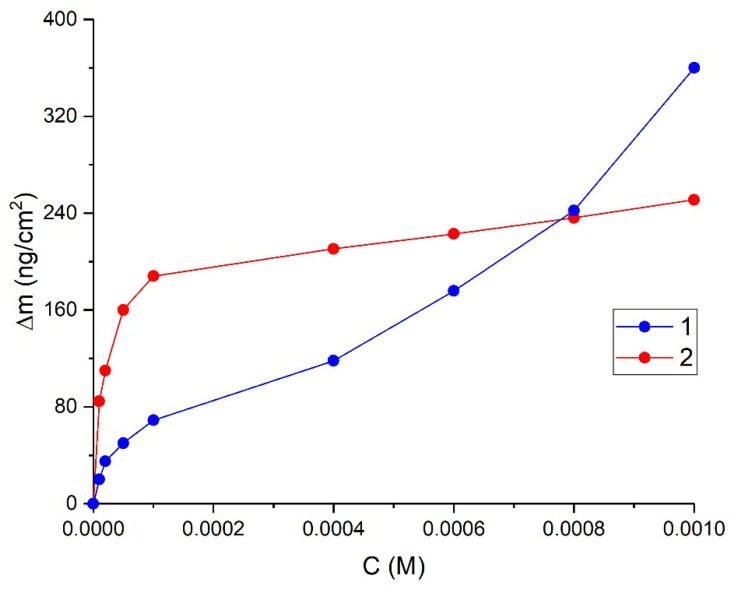
Isotherm of irreversible adsorption of organosilanes on the surface of freshly sprayed aluminium from aqueous solution: (**1**) DAS; (**2**) VS.

Figure 2 shows the adsorption isotherms of DAS and VS in a wide range of concentrations.

At low concentrations of DAS and VS (up to 0.0001 M), the type of adsorption isotherm corresponded to the Langmür isotherm. This indicates that the organosilane molecules were adsorbed by the monolayer. The monolayer could be calculated from bond lengths, and was about 1.5 and 0.9 nm for DAS and VS, respectively.

With an increase in the concentration of DAS, the mass of the adsorbed substance increased, which indicated polymolecular adsorption, and the VS was the adsorbed monolayer.

### 3.2. FTIR Spectroscopy 

**Figure 3:**
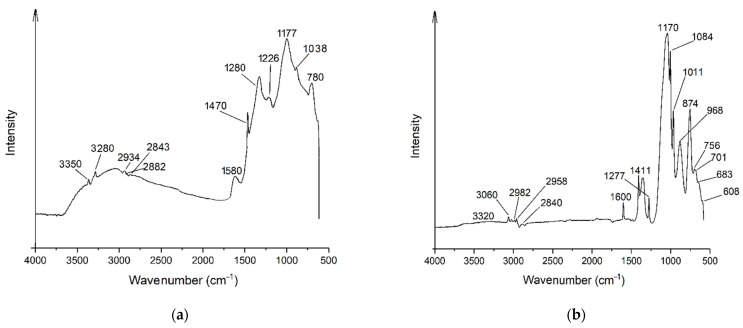
IR spectrums of films formed on the aluminium surface, after exposure metal samples for 60 min in organosilane solutions, in the region of 4000–500 cm^−1^: (**a**) DAS; (**b**) VS.

To explore the processes that occurred in the formation of organosilane films on metal, using Fourier-IR spectroscopy, we examined the surface of aluminium from the formed films from the used organosilanes solutions with a concentration of organosilanes of 0.1 M (Figure 3).

In Figure 3a, the IR spectrum for DAS contained a contour of the absorption bands of valence oscillations νNH (3350 and 3280 cm^−1^), as well as the maximum absorption bands of deformation oscillations δNH in the primary amino group (at 1580 cm^−1^). Differences could also be distinguished between oscillations of CH–groups: valence (νCH, maximum 2934 and 2882 cm^−1^) and strain (δCH_2_, 1470 cm^−1^). The absorption band of νCH in methoxy groups (at 2843 cm^−1^) was also recorded. The methoxysilane group was registered: the maximum absorption of oscillations 1177 cm^−1^ C–O, and 1038 cm^−1^ Si–O. Maximums at 1280, 1226, 1038 and 780 cm^−1^, corresponded to the absorption region of C–Si bonds. In the case of VS, because of the presence of a double bond, the properties of this organosilane changed significantly compared to DAS. As shown in the Figure 3b, there was an intense characteristic band of absorption of valence oscillations νCH from the methoxy group 2840 cm^−1^. The maxima at 2958 and 2982 cm^−1^ characterized the absorption of valence oscillations of the CH in several groups –O–CH_3_. The absorption band of hydroxyl groups had a slight intensity; protons at double bond gave low intensity absorption bands with maxima at 3060 and 3022 cm^−1^. An intense wide absorption band of associated OH–groups (at 3320 cm^−1^) was also recorded. In the IR spectrum of a VS, the absorption bands of the vinyl group (1600, 1411, 1011, 968 cm^−1^) and Si–O bonds (1084 cm^−1^) were recorded. Intense absorption bands at 1170, 874, and 756 cm^−1^ indicated C–O and Si–C bonds in the sample. During hydrolysis, VS on Al surface led to the breakage of the –O–CH_3_ bond with the formation of methanol groups –Si–OH, which formed covalent bonds with the metal. This was evidenced by the increase in absorption below 700 cm^−1^ in the infrared spectrum.

### 3.3. Structural Properties of Films

**Figure 4:**
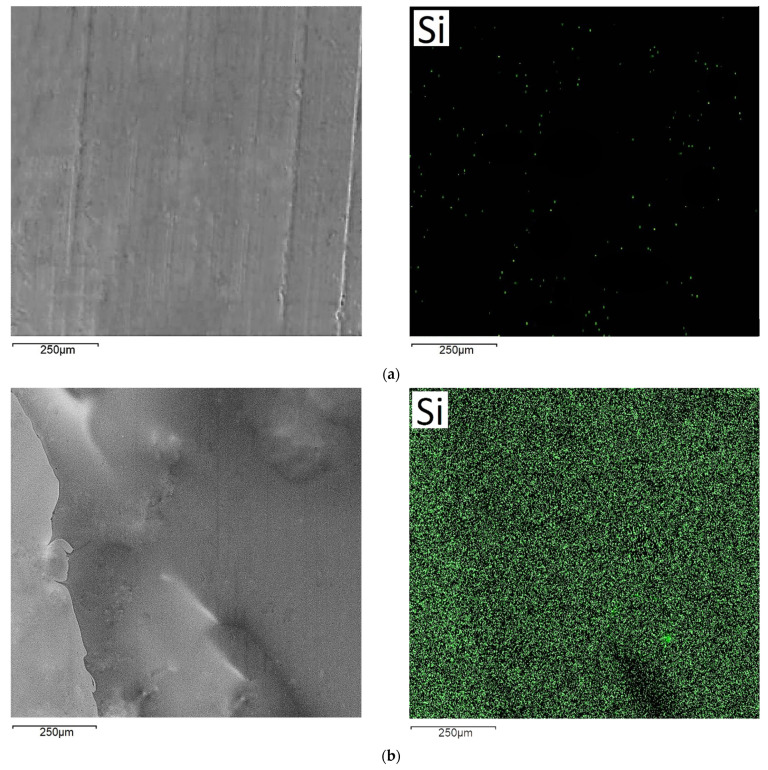

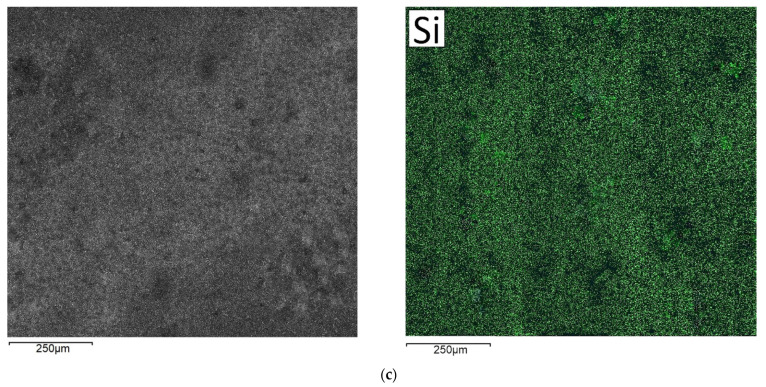
Electronic microphotography of clean aluminium surface and Al with organosilane films formed after 60 min of exposure to the modifying solution and the distribution of Si on the modified samples: (**a**) clean Al; (**b**) DAS; (**c**) VS.

SEM studies were carried out on aluminium samples after their exposure in organosilanes solutions for 60 min. Figure 4 shows electronic microphotography Al with organosilane films, as well as the distribution of silicon on the modified samples.

The SEM and EDS results are shown in Figure 4, which showed the uniform application of both organosilanes compared to the unmodified surface. This was evidenced by the silicon distribution map taken in the X-ray Kα line Si. Thus, the outer layer of the films consisted of siloxane groups that arose as a result of the polycondensation reaction. Subsequent exposure to temperature led to the formation of a denser structural network, similar to polymer films [8,9,11,26]. The thickness of the formed films for DAS was 1.2–1.9 μm, and for VS was 0.5–0.8 μm.

### 3.4. Corrosion–Electrochemical Behavior of Organosilane Films on the Surface of Al

**Figure 5:**
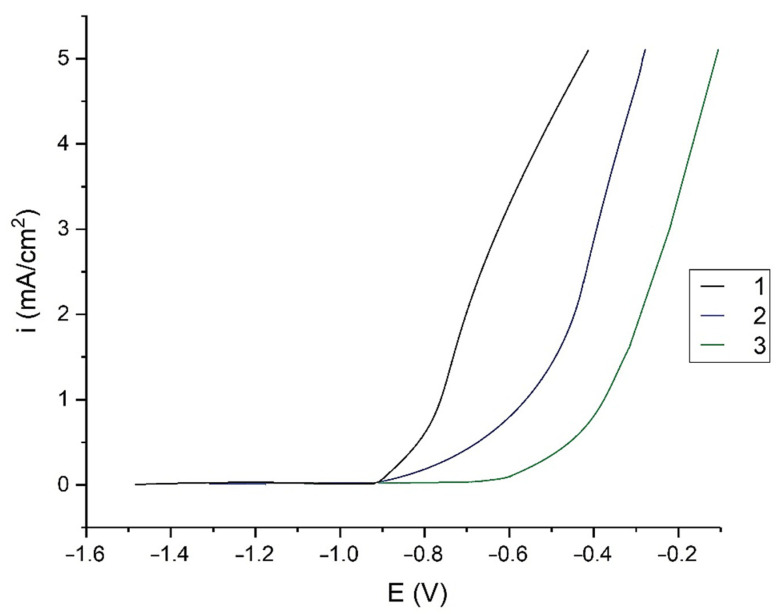
Anode polarization curves with formed organosilane films on the surface of aluminium and clean metal in a solution of 0.4 M H_3_BO_3_ + 0.1 M Na_2_B_4_O_7_ + 0.01 M NaCl pH 6.7: (1) clean Al; (2) Al + VS; (3) Al + DAS.

To assess the protective properties of the organosilane films formed on the Al surface, electrochemical studies and corrosion tests were carried out (Table 1; Figure 5).

From the data presented in Table 1, it can be seen that the presence of organosilane films on aluminium samples led to displacement of the corrosion potential in the anode region by 0.11 and 0.35 V for VS and DAS, respectively. In addition, the presence of vinyltrimethoxysilane film on the aluminium sample led to a displacement of the local depassivation potential by 70 mV, and in the case of a diaminsilane film $\Delta E_{pit\;}\;$= 180 mV. 

**Figure 6:**
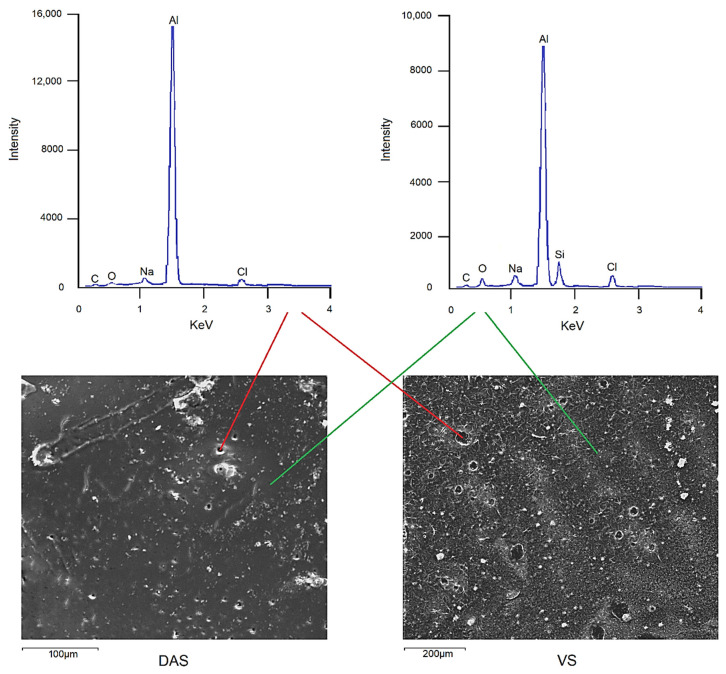
Microphotography of diaminsilane and vinyltrimethoxysilane films and EDS spectra on the surface of Al after the influence of anodic polarization in a solution of 0.4 M H_3_BO_3_ + 0.1 M Na_2_B_4_O_7_ + 0.01 M NaCl pH 6.7.

The results of electron microscopy of aluminium samples with vinyl- and diaminsilane films are presented in Figure 6.

**Figure 7:**
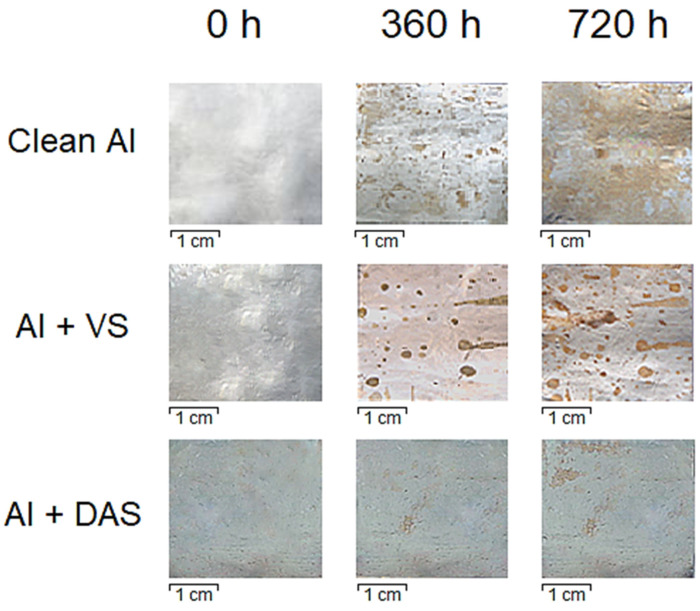
Photographs of the appearance of clean surface of Al and modified with organosilane films, after their exposure in the climatic chamber for 720 h.

Figure 6 showed that after the influence of anodic polarization, organosilane films were preserved on the metal surface. After removing the anode polarization curves in the films, there were defects in the form of pores. Therefore, in the case of VS, the film had a loose structure, and the maximum pore size was 15 μm. In the case of DAS, the film adhered more evenly to the aluminium surface, and the pore size was 9 μm. The results of the corrosion tests are presented in Figure 7 and Table 2.

Figure 7 demonstrated that after 720 h of exposure of samples in the climate chamber, local defect formation was recorded on the surface of all materials. A comprehensive assessment of the results presented in Table 2 showed that the system Al + DAS exhibited the best efficiency in these conditions. This was consistent with the previously obtained electrochemical results. Intermediate results of the system Al + DAS after 360 h of exposure showed a value of 0.1%, which was 10 times better compared to the system Al + VS. Furthermore, in descending corrosion resistance, it was possible to arrange the systems as Al + VS and clean aluminium.

**Figure 8:**
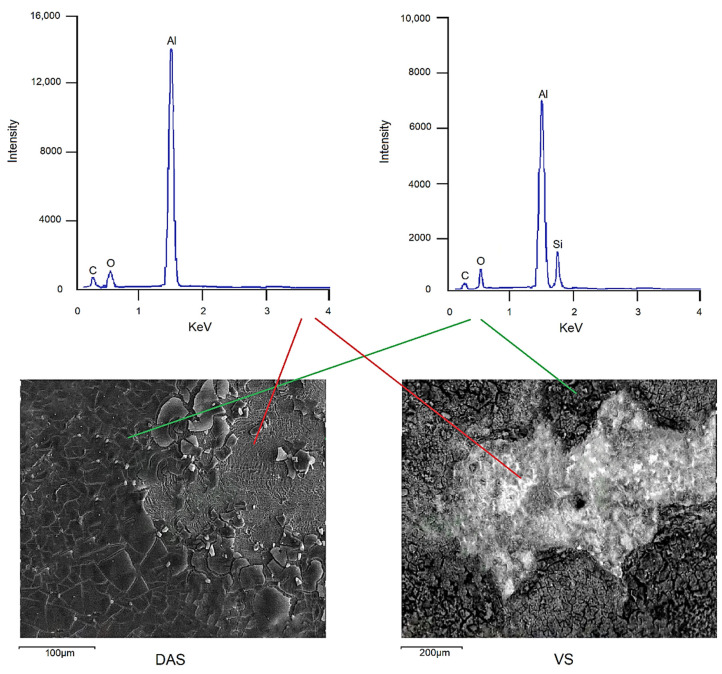
Microphotography of diaminsilane and vinyltrimethoxysilane films and EDS spectra on the surface of Al after 720 h of exposure in the climatic chamber at a temperature of 60 °C and humidity of 98%.

After exposure to anodic polarization and exposure of the samples in the climatic chamber, morphological studies of the films were carried out. Electron microscopy and EDS analysis data for aluminium samples with vinyl- and diaminsilane films after 720 h in a climate chamber are shown in Figure 8.

From the results presented in Figure 8, it can be seen that after 720 h of exposure of the samples in the climatic chamber, for both organosilanes, the films were present on the surface of the aluminium, but they were defective. In places where local foci of corrosion had arisen, the absence of films was recorded. This indicated a deterioration in anticorrosion properties. 

In general, on the surface of the samples on which organosilane films were formed, it was found that for DAS, places of detachment of the film from aluminium were much less. This explained the decrease in the proportion of corrosion lesions by an order of magnitude when compared with VS.

## 4. Discussion of Results 

### 4.1. Absorption of DAS and VS on the Surface of Aluminium

When processing adsorption data using different adsorption isotherms, adsorption heats were calculated for DAS and VS on the aluminium surface. The data obtained are shown in Table 3. 

As shown in Table 3, the heats of adsorption ranged from −25 to −47 kJ/mol, which exceeded the values recorded for the specific adsorption of organic compounds on metals. However, it is believed that chemisorption corresponds to the value of −100 kJ/mol, which was determined by the adsorption of thiols on gold [32]. At the same time, it is believed [33] that the value of the heat of replacement adsorption determined by quartz nanobalance has been greatly underestimated. This is because the energy of the interaction of water displaced from the surface on metals with a high over voltage on hydrogen is much higher than that on noble metals [2,32]. Thus, we can state that both DAS and VS are chemisorbed on the surface of aluminium.

### 4.2. Properties of Organosilane Films Formed on Al

**Scheme 1:**
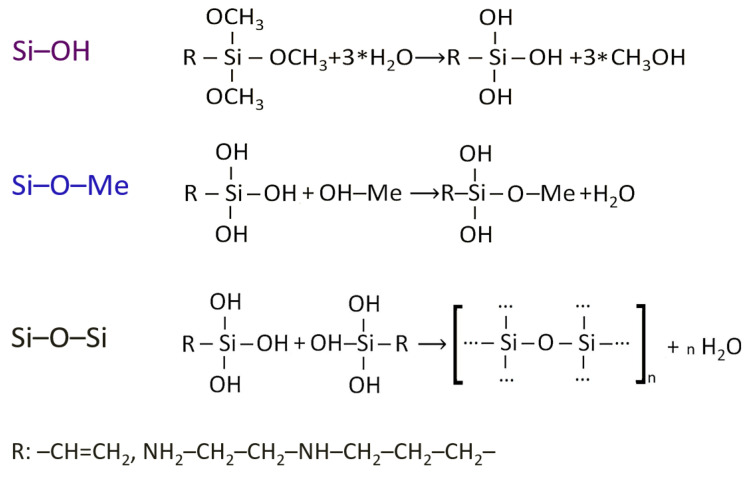
Reactions of the formation of organosilane films on the surface of Al.

The results of IR analysis, SEM, and EDS investigations of the modified surface of aluminium allow us to propose a scheme for the formation of organosilane films (Scheme 1) This confirms the previously proposed mechanisms for the formation of such films on the surfaces of iron, steel, zinc, and copper [7,8,15,20,21,22,26,34,35].

Once in the water, first, hydrolysis of molecules of organosilanes occurs with the formation of silanols ≡Si─OH. In parallel, condensation of silanol and surface hydroxyl groups of the metal occurs with the formation of surface metal–siloxane bonds ≡Si─O─Me. Finally, condensation of neighbouring silanol groups occurs to form the siloxane oligomer ≡Si─O─Si≡. The degree of oligomerisation n can vary from two to several hundred groups per square nanometer. In this case, given the density of hydroxyl groups on the surface and lengths of the Si–O bonds equal to 1.61 Å, n is in the range of 4–18 groups/nm^2^ [27]. Thus, summarizing the above experimental data, it can be assumed that the outer layers of organosilane films have polymer-like structures. Similar data are presented in the literature [8,26].

The results of this study of the corrosion–electrochemical behaviour of organosilane films on the surface of aluminium (anode polarization and exposure of samples in the climatic chamber for 720 h) showed the effectiveness of the diaminsilane films. Using data obtained from scanning electron microscopy, which also confirmed the effectiveness of the system Al + DAS due to the better continuity and uniformity of the distribution of the film of diaminsilane on aluminium, it can be assumed that this effect is due to the fact that: 1. DAS is highly soluble in aqueous media, i.e., it is a more hydrophilic organosilane compared to VS; and 2. diaminsilane, in its structure, has two amino-groups, which are catalysts for hydrolysis and polycondensation, contributing to the formation of a denser film uniformly coating the metal surface and improving the protective properties.

## 5. Conclusions

Applying piezoquartz nanoweights as well as IR-spectroscopy, it was found that the VS and DAS molecules were chemisorbed on the aluminium surface. Various adsorption models were used to calculate the adsorption heats for both organosilanes on aluminium. It was determined that at the initial stage of the formation of organosilane films on Al, the VS molecules are chemisorbed monolayer, and the DAS molecules are chemisorbed polymolecularly. Exposure of aluminium samples to aqueous solutions of organosilanes for 60 min, led to the formation of vinyltrimethoxysilane and diaminesilane films on its surface, 0.5–0.8 μm and 1.2–1.9 μm thick, respectively. Electrochemical and corrosion test results demonstrated that the presence of organosilane films on Al led to the inhibition of its dissolution. Thus, for VS, the local depassivation potential was 70 mV and for DAS was 180 mV. The exposure of metal samples with films for 720 h in the climatic chamber illustrated the best protective capacity of the Al + DAS system. For this system, the proportion of corrosion damage was 3% and the time before the appearance of the first defect was 242 h, whereas for Al + VS, it was 10% and 218 h. This result can be explained by the higher hydrophilicity of DAS compared to VS due to the presence of two NH-groups, which promoted better chemisorption of diaminosilane molecules on Al and, as a consequence, formation of a more complete and protective film on its surface. 

## Figures and Tables

**Table 1:** E_cor_ and E_pit_ clean and modified aluminium in organosilane solutions, in buffered chloride-containing solution.

System	E_cor_, V	E_pit_, V
Clean Al	−1.50	−0.94
Al + VS	−1.39	−0.87
Al + DAS	−1.15	−0.76

**Table 2:** The proportion of corrosion damage Q and time to occurrence of the first defect τ on clean and modified aluminium organosilane films during exposure of samples in the climatic chamber for 720 h.

System	τ, h	Q, %		
		Exposure Time, h		
		0	360	720
Clean Al	156	-	16.0	50.0
Al + VS	218	-	3.0	10.0
Al + DAS	242	-	0.3	3.0

**Table 3:** Adsorption isotherms to describe the adsorption of organosilanes from diluted aqueous solutions.

No.	Adsorption Isotherm	DAS		VS	
		Correlation, *R^2^*	$-\Delta G_{ads}^{{}^{\circ}},\;\mathrm{kJ}/\mathrm{mol}$	Correlation, *R^2^*	$-\Delta G_{ads}^{{}^{\circ}},\;\mathrm{kJ}/\mathrm{mol}$
1	Langmür	0.990	-	0.991	-
2	BET	1.000	−27.56	1.000	−25.48
3	Temkin	0.995	−47.32	0.923	−46.61
4	Langmür Multicenter	0.999	−45.15	0.999	−44.54
5	Flory–Huggins	0.999	−43.29	0.998	−40.73
6	Frumkin	0.994	−38.26	0.984	−37.96

## Data Availability

Not applicable.
